# Propofol Affects Optic Nerve Sheath Diameter less than Sevoflurane during Robotic Surgery in the Steep Trendelenburg Position

**DOI:** 10.1155/2019/5617815

**Published:** 2019-12-14

**Authors:** Yanghyun Kim, Seomun Choi, Sungwoo Kang, Boram Park

**Affiliations:** ^1^Department of Anesthesia and Pain Medicine, National Cancer Center, Gyeonggi-do, Republic of Korea; ^2^Biostatistics Collaboration Team, Research Core Center, Research Institute, National Cancer Center, Gyeonggi-do, Republic of Korea

## Abstract

**Background:**

Robot-assisted laparoscopic radical prostatectomy (RLRP) can increase intracranial pressure (ICP) related to a change in position. Increasing ICP may result in various ocular complications, which are rare but serious, such as a corneal abrasion and ischemic optic neuropathy. We performed a prospective observational trial using ultrasonographic measurements to compare optic nerve sheath diameter (ONSD) related to ICP between patients who received either propofol or sevoflurane and underwent RLRP.

**Methods:**

Thirty-two male patients scheduled to undergo RLRP were assigned into groups according to the anesthetic agent used (group P: propofol, *n* = 16; group S: sevoflurane, *n* = 16). ONSD, end-tidal partial pressure of CO_2_, and blood pressure were measured 10 min after induction of anesthesia (T0), 30 min (T1), 60 min (T2), and 90 min after changing to the steep Trendelenburg position and introducing a pneumoperitoneum (T3) and 10 min after returning the patient's position to supine (T4) during surgery.

**Results:**

No significant differences were observed in the demographic data of the patients, surgery time, or intraoperative variables, including hemodynamic and respiratory variables, at any of the time points. The mean right ONSDs in the propofol and sevoflurane groups were 37.3 and 40.1 mm at 30 min (*p*=0.003), respectively. The mean left ONSDs were 38.4 and 40.8 mm at 30 min (*p*=0.021) after changing to the Trendelenburg position. The ONSDs between the two groups were significantly different during surgery.

**Conclusions:**

ONSD increased more in the sevoflurane group than in the propofol group during RLRP. Intravenous anesthetics could alleviate the increase in ICP during RLRP.

## 1. Introduction

Prostate cancer is the second most common cancer in men worldwide. Robot-assisted laparoscopic radical prostatectomy (RLRP) has become a very popular treatment because it has better outcomes, less blood loss, and decreased recovery time compared to other options [1]. However, it requires patients to be in a steep Trendelenburg position, and an artificial pneumoperitoneum is introduced to facilitate surgical exposure by displacing the bowel cephalad. Together, these may result in various ocular complications, such as corneal abrasions and ischemic optic neuropathy (ION) [2]. The pathophysiology of ION remains unknown, although increased intraocular pressure is considered to be the cause [3].

Measuring optic nerve sheath diameter (ONSD) by ultrasonography is a very useful method for evaluating intracranial pressure (ICP); it is strongly correlated with invasive ICP measurements estimated using intraventricular and intraparenchymal devices [4].

All inhaled anesthetics have a direct, dose-dependent vasodilator effect on the cerebral vessels that increases cerebral blood volume and blood flow [5]. Sevoflurane has the least effect among other comparable agents [6, 7]. Propofol decreases ICP but maintains cerebral perfusion pressure [8].

In the present study, we hypothesized that ONSD in the sevoflurane group would increase more than that in the propofol group.

## 2. Methods

This study had a prospective, randomized, double-blind design and was approved by the Institutional Review Board of the National Cancer Center (NCC2017-0065). It is registered with the Korean Clinical Trials Registry (CRiS, http://cris.nih.go.kr, 9/11/2018, KCT 0003332). After obtaining written informed consent, we enrolled 32 male patients between 19 and 79 years of age with an American Society of Anesthesiologists (ASA) physical status of I–III who were undergoing RLRP. Patients with a previous neurological disease or cerebrovascular disease that could increase ICP or patients with a history of ophthalmological disease or surgery were excluded.

After preoxygenation, anesthesia was induced with remifentanil and propofol followed by rocuronium (0.6 mg/kg) to facilitate tracheal intubation. After endotracheal intubation, the patients were mechanically ventilated with a 50% oxygen-air mixture, using a tidal volume of 8 mL/kg ideal body weight at a respiratory rate of 10–14/min to maintain end-tidal CO_2_ partial pressure (ETCO_2_) between 35 and 40 mmHg. The random assignments were generated by using a computer with block sizes of 2 and 4 with a 1 : 1 assignment using the random block size permutation method. Patients were randomized into two groups: group S or group P. In group S, anesthesia was maintained with 1-2 vol% sevoflurane and an infusion of remifentanil. In group P, anesthesia was maintained with propofol and an infusion of remifentanil. The end-tidal concentration of sevoflurane or propofol was adjusted to maintain a bispectral index score of 40–60, and the targeted effect-site concentration of remifentanil was adjusted to 1.0–3.0 ng/mL using a target-controlled infusion (TCI) device.

**Figure 1:**
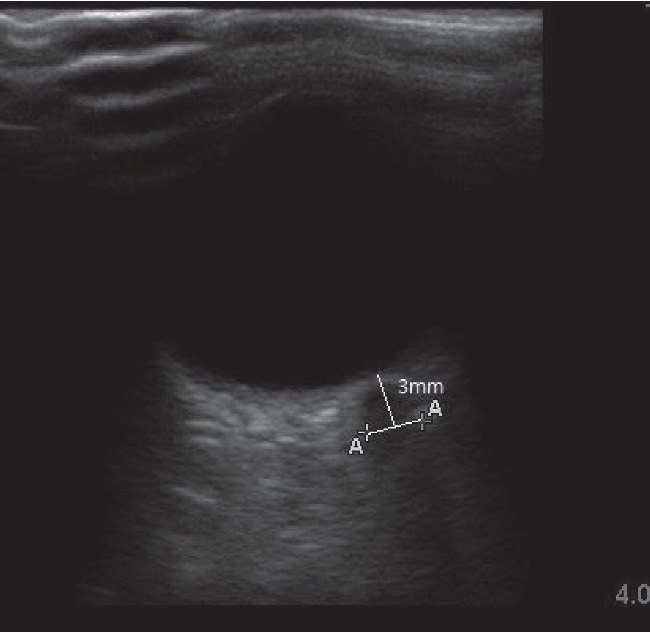
Optic nerve sheath sonography.

The vaporizer and TCI pump were concealed from the anesthesiologist by using a screen. The blinded anesthesiologist measured ONSD, evaluated as described previously [9]. Briefly, the patients were placed in the supine position or steep Trendelenburg position with their eyelids taped closed, and a thick layer of gel was applied to their closed upper eyelids. A 7.5 MHz linear probe (M-turbo™ ultrasound system, Sonosite, USA) with reduced acoustic power was applied on the gel without excessive pressure to avoid damaging the retina and lens, and the ultrasound beam was adjusted to provide a suitable angle for displaying the entry of the optic nerve into the globe to measure ONSD. The ONSD was measured 3 mm behind the optic disc (Figure 1), at five time points: 10 min after induction of anesthesia (T0), 30 min after changing to the steep Trendelenburg position and introducing a pneumoperitoneum (T1), 60 min after changing to the steep Trendelenburg position and introducing a pneumoperitoneum (T2), 90 min after changing to the steep Trendelenburg position and introducing a pneumoperitoneum (T3), and 10 min after returning the patient's position to supine (T4). Each measurement was conducted three times on each orbit in the transverse plane of both eyes. The baseline ONSD was measured 10 min after induction. We used the mean of three values at each point for analyses.

ETCO_2_, blood pressure, and heart rate (HR) were measured at each time point. We also measured surgery time and the volume of infused fluid. In pilot study, the mean standard deviation of ONSD was 38.83 ± 2.23 mm in the sevoflurane group and 36.17 ± 3.31 mm in the propofol group. The difference was about 3 mm in the pilot study. However, the difference of about 10% was considered clinically significant. We assumed that the mean difference was 4.0 mm for the primary endpoint of ONSD at 30 min after changing to the Trendelenburg position with a standard deviation in both groups of 2.8. We calculated that 14 patients in each group would achieve 95% power to reject the null hypothesis of equal means with a significance level of 0.05 using a two-sided two-sample equal-variance *t*-test. We recruited 16 patients per group, considering a dropout rate of 10%. The primary outcome in this study was the group difference in ONSD at 30 min after the head-down position. The secondary outcomes included were as follows: the group difference in ONSD at 10 min after induction of anesthesia, 60 min and 90 min after head-down position, and 10 min after supine position; end-tidal carbon dioxide pressure; blood pressure at 10 min after induction of anesthesia, 30 min, 60 min, 90 min after head-down position, 10 min after supine position and; infused intravenous fluid at the end of surgery.

The independent *t*-test was used to evaluate the difference between the propofol and sevoflurane group in regard to changes in ONSD, systolic blood pressure, diastolic blood pressure, mean blood pressure, HR, and ETCO_2_. The difference between the time points in each group was tested with the paired *t*-test, and the differences between the two groups over time were compared based on a mixed-effect model. All statistical analyses were performed using SAS version 9.4 (SAS Institute Inc., Cary, NC, USA).

## 3. Results

Thirty-two patients scheduled for RLRP were included and evaluated. The patients' characteristics are shown in Table 1. No significant differences were observed in the demographic data of the patients, surgery time, or intraoperative variables, including hemodynamic and respiratory variables at any of the time points.

**Figure 2:**
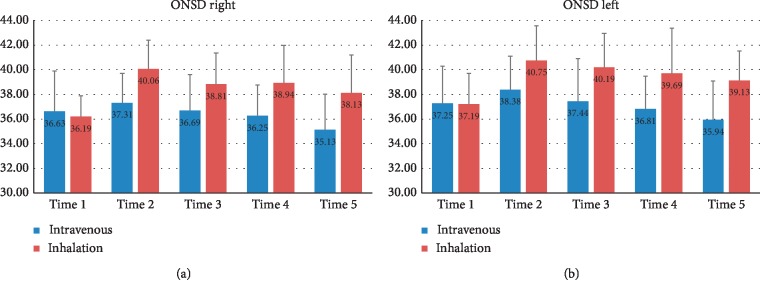
Mean and standard deviation optic nerve sheath diameters in the propofol and sevoflurane groups.

ONSD was measured at 10 min after induction of anesthesia (T0), 30 min (T1), 60 min (T2), and 90 min (T3) after changing to the Trendelenburg position and introducing a pneumoperitoneum, and 10 min after the position changes to supine (T4) in Table 2. The mean right ONSDs in the propofol and sevoflurane groups were 36.6 and 36.2 mm (*p*=0.638) at T0, 37.3 and 40.1 mm (*p*=0.003) at T1, 36.7 and 38.8 mm (*p*=0.036) at T2, 36.3 and 38.9 mm (*p*=0.011) at T3, and 35.1 and 38.1 mm (*p*=0.008) at T4, respectively. The mean left ONSDs were 37.3 and 37.2 mm (*p*=0.950) at T0, 38.4 and 40.8 mm (*p*=0.021) at T1, 37.4 and 40.2 mm (*p*=0.019) at T2, 36.8 and 39.7 mm (*p*=0.017) at T3, and 35.9 and 39.1 mm (*p*=0.003) at T4 (Figure 2) The ONSDs between the two groups were significantly different during surgery (Table 3). ONSD in the sevoflurane group were increased significantly from 36.2/37.2 mm (right/left) at baseline to 40.1/40.8 mm (right/left) 30 min after the postural change (*p* < 0.01). ONSDs of the propofol group were significantly increased only in the left from 37.3 (T0) mm to 38.4 mm (T1) (*p*=0.042).

## 4. Discussion

We investigated changes in ONSD according to the anesthetic agent. We observed significant differences between the sevoflurane and propofol groups. Whitely et al. [10] reported a significant increase in ONSD of patients undergoing RLRP. However, few studies have reported how specific anesthetic agents affect the change in ONSD. ONSD did not change over time in the propofol group. However, it increased significantly 30 min after the postural change in the sevoflurane group but did not increase 60 or 90 min after changing to the Trendelenburg position and inducing a pneumoperitoneum. These results are consistent with previous studies [11]. Verdonck et al. [12] described a compensatory mechanism for a shift in cerebrospinal fluid towards the spinal canal and the vascular compartment.

Measuring ONSD is a noninvasive method that correlates significantly with changes in ICP [12]. ONSD values > 5 mm are associated with ICP values > 20 mmHg [13, 14]. We did not measure ONSD values > 5 mm or observe any postoperative adverse neurological sequelae in our study.

A prolonged steep Trendelenburg position and increasing intra-abdominal pressure can result in increased cerebral venous pressure, ICP, and cerebral blood flow. Weber et al. [15] reported postoperative visual loss due to a prolonged steep Trendelenburg position during minimally invasive prostatectomy. Molloy [16] reported that intraocular pressure increases overtime in the steep Trendelenburg position. Ocular perfusion pressure is calculated as arterial pressure minus IOP. Therefore, when intraocular pressure rises, ocular perfusion pressure decreases and can cause an increased risk of postoperative visual loss.

ICP can be affected by carbon dioxide, blood pressure, infused fluid, and operation time [17]. In our study, no significant differences were observed in the parameters that affect ICP between the two groups.

Many studies have demonstrated that sevoflurane has a dose-dependent cerebral vasodilatory effect and that propofol either decreases or does not affect ICP. In our study, ONSD did not increase in the propofol group. We hypothesized that the use of propofol would not affect ICP in patients at risk for elevated ICP, such as those in the steep Trendelenburg position, and would not increase ONSD. We confirmed this in our study.

Some limitations of this study should be mentioned. We only measured PaCO_2_ at baseline, which may have affected ICP. However, because EtCO_2_ has a strong relationship with PaCO_2_, we maintained EtCO_2_ partial pressure at 35–40 mmHg over the whole period and adjusted ventilator settings in both groups. No significant difference in EtCO_2_ was detected between the two groups.

In conclusion, ONSD during RLRP increased in the sevoflurane group but not in the propofol group. Intravenous anesthetics could alleviate the increase in ICP during RLRP. We recommend using intravenous anesthetics when performing this surgery in a position that could cause an elevation in ICP, such as the prolonged steep Trendelenburg position and when placing an artificial pneumoperitoneum, because sevoflurane has a cerebral vasodilatory effect although a sufficient compensatory mechanism may weaken the intracranial effects.

## Figures and Tables

**Table 1:** Baseline characteristics.

Characteristics		Total	Propofol group	Sevoflurane	*p* value
		(*n* = 32)	(*n* = 16)	(*n* = 16)	
Age		66.41 ± 8.05	64.38 ± 7.86	68.44 ± 7.97	0.159
Height		167.28 ± 5.16	167.63 ± 5.94	166.94 ± 4.42	0.713
Weight		68.03 ± 9.43	69.38 ± 10.25	66.69 ± 8.65	0.429
ASA	1	6 (18.75)	4 (25.00)	2 (12.50)	0.654
	2	26 (81.25)	12 (75.00)	14 (87.50)	
Operation time		182.19 ± 31.18	183.13 ± 32.91	181.25 ± 30.41	0.868
Anesthesia time		229.69 ± 33.72	231.88 ± 35.68	227.5 ± 32.66	0.720
Infused fluid		1298.44 ± 421.11	1287.5 ± 373.05	1309.38 ± 476.52	0.886

End-tidal CO_2_					
T0		31.16 ± 2.64	32.06 ± 2.62	30.25 ± 2.41	0.051
T1		33.63 ± 2.11	33.75 ± 2.41	33.50 ± 1.83	0.743
T2		33.44 ± 2.46	33.25 ± 2.98	33.63 ± 1.89	0.674
T3		34.59 ± 2.66	34.25 ± 3.17	34.94 ± 2.08	0.474
T4		31.94 ± 3.12	31.63 ± 3.44	32.25 ± 2.84	0.580

Systolic blood pressure					
T0		109.69 ± 14.91	110.13 ± 15.88	109.25 ± 14.39	0.871
T1		117.66 ± 17.74	125.00 ± 19.90	110.31 ± 11.80	0.017
T2		115.03 ± 17.66	118.38 ± 19.47	111.69 ± 15.56	0.292
T3		112.13 ± 15.73	116.44 ± 15.19	107.81 ± 15.52	0.123
T4		132.25 ± 18.77	129.19 ± 17.28	135.31 ± 20.24	0.365

Diastolic blood pressure					
T0		56.53 ± 9.13	56.56 ± 7.36	56.5 ± 10.88	0.985
T1		66.75 ± 8.82	68.81 ± 8.89	64.69 ± 8.52	0.190
T2		66.88 ± 9.85	68.13 ± 10.07	65.63 ± 9.78	0.482
T3		66.16 ± 10.12	68.38 ± 11.00	63.94 ± 8.96	0.221
T4		68.72 ± 11.43	67.63 ± 10.22	69.81 ± 12.76	0.597

Ten minutes after inducing anesthesia (T0), 30 min after changing to the Trendelenburg position and introducing a pneumoperitoneum (T1), 60 min after changing to the Trendelenburg change and introducing a pneumoperitoneum (T2), 90 min after changing to the Trendelenburg position and introducing a pneumoperitoneum (T3), and 10 min after discontinuing the pneumoperitoneum and the patient's position was returned to supine (T4).

**Table 2:** Comparison of optic nerve sheath diameter between intravenous and inhalation anesthesia.

Characteristics	Total	Propofol	Sevoflurane	*p* value
	(*n* = 32)	(*n* = 16)	(*n* = 16)	
ONSD (Rt)				
T0	36.41 ± 2.56	36.63 ± 3.26	36.19 ± 1.68	0.638
T1	38.69 ± 2.71	37.31 ± 2.39	40.06 ± 2.32	0.003
T2	37.75 ± 2.90	36.69 ± 2.91	38.81 ± 2.54	0.036
T3	37.59 ± 3.07	36.25 ± 2.52	38.94 ± 3.04	0.011
T4	36.63 ± 3.30	35.13 ± 2.90	38.13 ± 3.05	0.008

ONSD (Lt)				
T0	37.22 ± 2.73	37.25 ± 3.02	37.19 ± 2.51	0.950
T1	39.56 ± 2.97	38.38 ± 2.73	40.75 ± 2.79	0.021
T2	38.81 ± 3.38	37.44 ± 3.46	40.19 ± 2.76	0.019
T3	38.25 ± 3.47	36.81 ± 2.66	39.69 ± 3.66	0.017
T4	37.53 ± 3.18	35.94 ± 3.13	39.13 ± 2.39	0.003

ONSD, optic nerve sheath diameter.

**Table 3:** Differences over time within each group.

				*p* value
Propofol	ONSD (Rt)			
		T1	37.31 ± 2.39	0.214
		T0	36.63 ± 3.26	
		T1	37.31 ± 2.39	0.098
		T2	36.25 ± 2.52	
	ONSD (Lt)			
		T1	38.38 ± 2.73	0.042
		T0	37.25 ± 3.02	
		T1	38.38 ± 2.73	0.030
		T2	36.81 ± 2.66	
Sevoflurane	ONSD (Rt)			
		T1	40.06 ± 2.32	<0.0001
		T0	36.19 ± 1.68	
		T1	40.06 ± 2.32	0.169
		T2	38.94 ± 3.04	
	ONSD (Lt)			
		T1	40.75 ± 2.79	<0.0001
		T0	37.19 ± 2.51	
		T1	40.75 ± 2.79	0.059
		T2	39.69 ± 3.66	

ONSD, optic nerve sheath diameter.

## Data Availability

The data used to support the findings of this study are included within the article.
